# Pulsed Light (PL) Treatments on Almond Kernels: *Salmonella enteritidis* Inactivation Kinetics and Infrared Thermography Insights

**DOI:** 10.1007/s11947-021-02725-9

**Published:** 2021-11-04

**Authors:** Maitê Harguindeguy, Carlos E. Gómez-Camacho

**Affiliations:** DISAT, Dep. Applied Science and Technology, Politecnico Di Torino, C/so duca degli Abruzzi 24, 10129 Torino, Italy

**Keywords:** Pulsed light, Infrared thermography, *Salmonella enteritidis*, Almonds, Nuts, Photothermal, Weibull, Bigelow, Microbial inactivation, Non-thermal processing

## Abstract

**Abstract:**

Extending the shelf-life and ensuring microbiological safety of food products while preserving the nutritional properties are key aspects that must be addressed. Heat processing of food matrices has been the golden standard during the last decades, while certain non-thermal processing options have recently gained ground. In the present study, experimental pulsed light (PL) surface inactivation treatments of *Salmonella enteritidis* on almonds kernels are performed. The PL system is set to test different operative conditions, namely power (1000, 1250, and 1500 W) and frequency (1.8, 3.0, and 100.0 Hz) at different treatment times (from 5 to 250 s), which result in applied fluence doses in the 0–100 J·cm^−2^ range. Additionally, temperature measurements are collected at each operative condition on the almond surface (using infrared (IR) thermography) and at the superficial layer of the almond (1-mm depth using a thermocouple). The observed PL inactivation kinetics are then modelled using four different models. The best goodness-of-fit is found for the two-parameter Weibull model (*R*^2^ > 0.98 and RMSE < 0.33 for all cases). The maximum achieved log-CFU reductions are 6.02 for the 1.8-Hz system, 4.69 for the 3.0-Hz system, and 3.66 for 100.0-Hz system. The offset between the collected temperature readings by the two sensors is contrasted against the inactivation rate (following the two-parameter Weibull model). It was found that the highest inactivation rate corresponds approximately to the point where the infrared camera detects a slowdown in the surface heating.

**Graphical Abstract:**

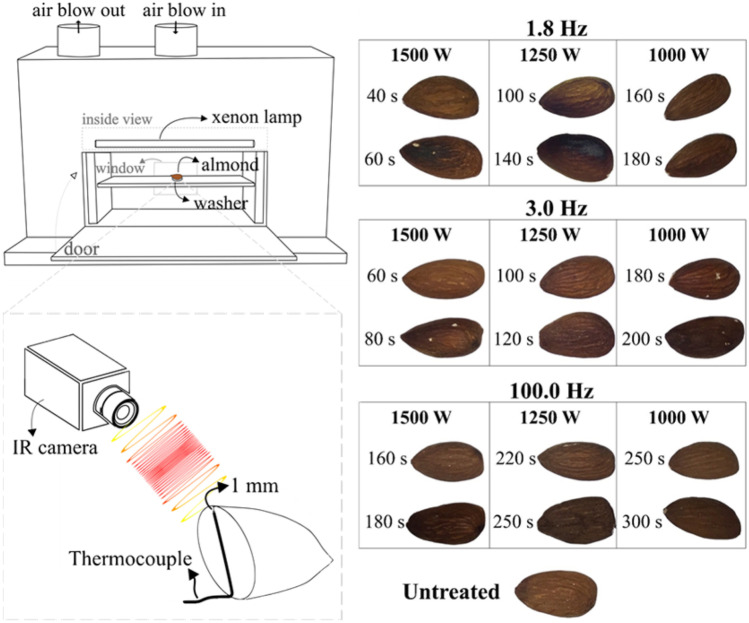

**Supplementary Information:**

The online version contains supplementary material available at 10.1007/s11947-021-02725-9.

## Introduction

According to World Health Organization (WHO), over 200 diseases are caused by food poisoning. Microbial pathogens such as bacteria, viruses, and parasites and exposure to certain chemical substances such as heavy metals are the main food contaminants. The contamination of food matrices occurs not only during the food production stage, but also in the transportation and consumption stages. Among the most common emerging and reemerging foodborne pathogens, the genera *Salmonella*, *Campylobacter*, *Escherichia,* and *Listeria* are frequently encountered (WHO, 2021). However, effectively controlling these pathogens on fresh products is also becoming exacerbated due to the natural adaptation capacity of microorganisms.

Thermal inactivation techniques have long been used to decontaminate different food matrices.They have proven their effectiveness through their well-described dynamics that can be easily modeled and reported in terms of the *Z* and *D* values. Depending on the applied doses and the nature of the heat shock (i.e., dry or moist heat), heat inactivation can potentially act on vegetative and dormant cells, as well as on heat-labile toxins. Nevertheless, there are still periodic outbreaks associated with foodborne diseases worldwide, partly due to the natural microbial adaptation capabilities. In particular, the number of foodborne outbreaks associated with the consumption of fresh products (e.g., fruits, vegetables, nuts, eggs) has significantly increased during the last years (Farakos et al., 2017; Pijnacker et al., 2019).

The production of almonds in 2019 amounted to 3.5 million tons worldwide; the USA accounts for more than 54% of the global production representing a 6-billion-dollar industry (Economic Research Service, 2017). Other countries such as Spain, Iran, Turkey, Australia, Morocco, Syria, and Italy share more than 30% of the global production (FAO, 2020). Salmonellosis outbreaks involving almonds produced in the USA have been historically reported. In 2000 and 2001, raw almonds contaminated with *Salmonella enteritidis* (SE) phage type 30 (PT 30) resulted in 157 salmonellosis cases in Canada and 11 cases in the USA (Isaacs et al., 2005). Later, similar outbreaks in 2004, 2005, and 2006 reaffirmed the relevance of *Salmonella* contamination in raw almonds as some outbreaks resulted in deaths (Harris et al., 2015).

Different regulatory agencies have partly addressed this issue. For example, the US Department of Agriculture enacted in 2007 a dedicated quality control requirement (United States Department of Agriculture, 2007). This standard establishes that almonds must be treated by a process capable of achieving at least a 4-log *Salmonella *population reduction before commercialization. At the same time, the Almond Board of California sets guidelines on process validation for oil roasting, propylene oxide pasteurization, blanching, and dry air roasting (Almond Board of California, 2007a, 2007b; Harris, 2007a, 2007b). However, traditional almond processing technologies often adversely affect quality attributes such as color, freshness, appearance, and flavor; hence, improved alternative inactivation processes are always needed.

On the other hand, protective responses against inactivation agents are a common risk that cannot be disregarded while processing food matrices. For example, it has been observed that the *Salmonella* genus can develop an acid tolerance response (ATR), which has also been reported to provide cross-protection against heat inactivation. This fact is becoming critical in the food industry. Therefore, multiple inactivation agents or combined treatments are being tested to guarantee an adequate microbiological safety of fresh or processed foods. The efficacy, advantages, and disadvantages of alternative technologies for *Salmonella* inactivation on almonds surface, such as infrared heating, radio frequency heating, high-pressure processing, and electron beams, have been reviewed previously (Pan et al., 2012). Additionally, experimental insights have been gathered during the last years: Bingol et al. (2011) used *Pediococcus* spp. as a surrogate for *Salmonella* and obtained over 5-log CFU reductions by infrared heating without noticeable quality impairment. In other studies (Gao et al., 2011; Jeong et al., 2017), *Salmonella* inactivation on almond surfaces reached 5-log CFU using radio frequency heating at laboratory scale. Prakash et al. (2010) evidenced that a 4-log reduction for SE PT30 was possible by electron beam irradiation. However, the final product was sensorially not acceptable (Prakash et al., 2010). Meanwhile, Willford et al. (2016) obtained over 4-log reductions for SE PT30 using hydrostatic pressure processing (Willford et al., 2016). Another novel technology recently used for microbial inactivation in food matrices is pulsed light (PL) (Abida et al., 2014).

PL treatment typically entails the exposure of food matrices (solid, liquid, and semi-solid), food packaging materials, or surfaces to short-duration high-power pulses of light (i.e., in the 100–1000 nm range, from the UV to the near-infrared spectrum). Besides this large energy spectrum that characterizes PL, the effectiveness of the treatment seems to be driven by the greater and instantaneous energy impact compared to UV continuous light. Hence, the inactivation mechanism of PL tends to be described as a combination of photochemical, photophysical, and photothermal effects (Krishnamurthy et al., 2010). The photophysicochemical effect is attributable to the damage of cell components or structures. In contrast, the photothermal effect is attributable to the infrared (IR) spectrum, involving the fast dynamic phenomena related to heat transfer mechanisms that cells and the food matrices undergo.

The coexistence among these lethal effects has been widely hypothesized, while the relative importance of each is likely dependent on the fluence doses and the targeted microorganisms (Condon et al., 2014). Among the photophysicochemical effects, the damage associated with the UV spectrum of PL treatment has been found to derive from its interaction with the nucleic acids of the biotic phase. Indeed, the UV inactivation treatment operates by triggering the formation of thymine dimers within the microbial DNA. In turn, the formation of these dimers can potentially block vital cellular functions such as DNA transcription and replication, and ultimately, it can lead to cell death (Elmnasser et al., 2007; Kramer et al., 2017). Conversely, photothermal effects are believed to be generated due to the absorption of UV and IR radiations and the consequent dissipation into heat. This pulsed light-induced localized heating takes part in the complex global heat transfer balance. Other important factors of this balance are the heating and cooling rates, the absorption and moisture characteristics of the biotic phase, the food matrix, and the surrounding environment. Generally, the lethal photothermal effects are described as the result of the biotic phase acting as vaporization centers that might induce cell wall rupture.

PL processing can be a promising novel method for microorganism inactivation for several reasons. First, it requires short treatment times to achieve the desired inactivation levels, which are very compelling for commercial applications. Due to these short treatment times, this process usually has a reduced effect on the flavor and overall quality of foods (Elmnasser et al., 2007). Additionally, it is more effective in inactivating microorganisms than continuous UV light in most cases due to its high instantaneous power (Condon et al., 2014). Indeed, PL inactivation has proved effective for different microorganisms (e.g., bacteria, spores, mold, and viruses) in various food matrices such as milk (Krishnamurthy, 2006; Krishnamurthy et al., 2005), fruit juices (Preetha et al., 2021), cheese (Can et al., 2014), seafood (Lasagabaster & Martínez de Marañón, 2017), poultry (Keklik et al., 2011), pineapple juice (Vollmer et al., 2020), fresh fruits such as blackberries and strawberries (Bialka et al., 2008) and blueberries, black pepper (Xu, 2016), among others (Rowan, 2019). Recently, it was found to be a suitable candidate to inactivate SARS-CoV-2 in hospital environments (Jubinville et al., 2021).

Pulsed light treatments have been applied on hard food matrices for surface inactivation. For example, Öner (2017) tested PL on almonds and achieved *Salmonella* population reductions from 0.44 to 4.14 log CFU/almond. Liu et al. (2021) tested wetting almonds by one- or two-time water immersions and achieved 5-log reductions of *Salmonella.* In those studies, the almond heating was assessed using a thermocouple inside the treated almonds. But the inactivation kinetics was described in the time domain (instead of the fluence doses), and no inaction models were evaluated.

The present study aims to experimentally test PL surface inactivation treatments on *S. enteritidis* PT 30-inoculated almond kernels. The PL tests for the inactivation of *Salmonella* on almond kernel surfaces are applied at different operative conditions to gain insights into the performance of the treatments using applied fluence doses in the 0–100 J·cm^−2^ range. Different equipment components were used, allowing a wide range of conditions to be tested (Table 1). The present experimental design aims (i) to perform PL treatments and to identify process configurations that reach 4-log inactivation levels without compromising the stability of the food matrix, (ii) to verify the thermal effects of PL treatments on the selected food matrix using a tandem temperature measurement system, and (iii) to test the suitability of different mathematical models to describe the achieved bacterial inactivation.

### Material and Methods

#### Bacterial Inoculum, Almond Kernels, and Sample Preparation

A stock liquid culture of *S. enteritidis* PT 30 (ATCC BAA-1045) was prepared using tryptic soy broth (Difco TSB, Becton Dickinson, Sparks, MD) as the cultivation medium. This stock culture was incubated at 37 °C for 24 h, and it was then stored at 4 °C. Subsequently, petri dishes were inoculated with 100 µL aliquots of the liquid stock culture by spread plating this inoculum volume on tryptone soya yeast extract agar (Difco TSAYE, Sparks, USA) medium; samples were then incubated at 37 °C for 24 h. After this incubation step, the formed *Salmonella* culture was harvested by loosening the cells with sterile swabs on top of the petri dishes and resuspending them in 1 mL phosphate buffer saline solution (Fisher Scientific PBS, Pittsburgh, PA) at 0.5 M and pH = 7.4.

**Table 1:** Specifications of the tested PL control modules

Control module	Pulse frequency (Hz)	Capacitance (µF)	Maximum voltage (V)
1-C-115-H400	1.8	115	3800
1-C3-H50	3.0	70	3800
1-C70-H200	100.0	3	3600

Almond kernels (Fisher Whole Natural Almonds®, Modesto, USA) purchased from a local grocery store were used for the tests. The almonds’ superficial inoculation was performed by placing a 10 µL aliquot of the resuspended cells on the geometric center of the top surface of each almond. Then, these spot-inoculated almonds were kept inside closed sterile petri dishes for 24 h and ambient temperature (c. 22 °C) to allow the drying process and the adhesion of the cells to take place. The PL treatments were tested on these spot-inoculated samples; the initial inoculation level amounts to ~ 1 × 10^9^ CFU/almond (~ 0.68 × 10^9^ CFU/g almond).

#### Experimental Setup and Design of the PL Treatments

**Fig. 1:**
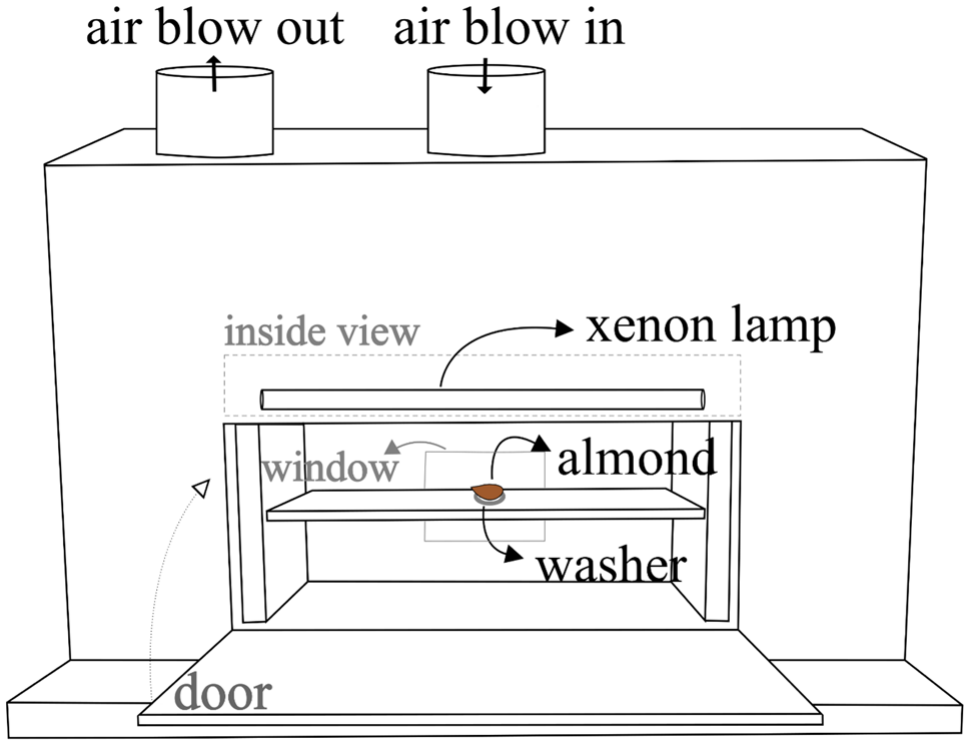
PL chamber used (not to scale). Drawing showing a schematic of the almond placement inside the chamber, the window through which the IR camera monitored the surface temperature and other system details

The selected PL processing unit was a modular UV curing system (Xenon RC-800 series, Wilmington, USA) equipped with three customizable control modules (see Table 1). This PL processing unit was connected to a sterile treatment chamber, which is equipped with a 16″ linear lamp (Xenon SteriPulse-XL, Wilmington, USA). The lamp was continuously cooled by an air blower (i.e., at a rate of approx. 300 ft^3^·min^−1^), and the entire setup was placed within an exhaust hood to remove residual ozone that might be produced during the PL treatment (see Fig. 1). The fluence dose delivered to the food matrix varies according to the inverse square law (i.e., the relative position of the sample with respect to the PL source) (Gómez-López & Bolton, 2016). To adequately describe the observed *Salmonella* inactivation levels in terms of the fluence dose, one almond was treated per experiment, each done with three repetitions.

The voltage of PL treatments units was adjusted utilizing the controller unit and a digital multimeter (Fluke 115 True-RMS, American Fork, USA). The target operative voltage for each module is calculated using Eqs. 1 and 2. In this set of equations, *P* is the electrical power (W), *V* is the voltage (V), *f* is the frequency (Hz), and *C* is the capacitance (F).

1 $$P =\frac{{V}^{2} \times C \times f}{2}$$

2 $$V=\sqrt{\frac{2 \times P}{C \times f}}$$

Three different power conditions were tested: 1000, 1250, and 1500 W. The energy per pulse varied due to the default pulse rate of each of them (i.e., 1.8, 3.0 and, 100.0 Hz) and the power conditions. In PL treatment systems, the alternate current is converted into high voltage direct current, and it is stored at high-density conditions in a capacitor prior to the pulsed lamp discharges. Within the lamp, the energy serves to ionize the contained inert gas thus promoting the emission of short intense light pulses. Whereas in general, the pulse duration can be as short as approx. 100 ns up to 2 ms, at fixed power conditions, the higher the pulse rate, the lower the energy contained in each pulse and vice versa.

**Fig. 2:**
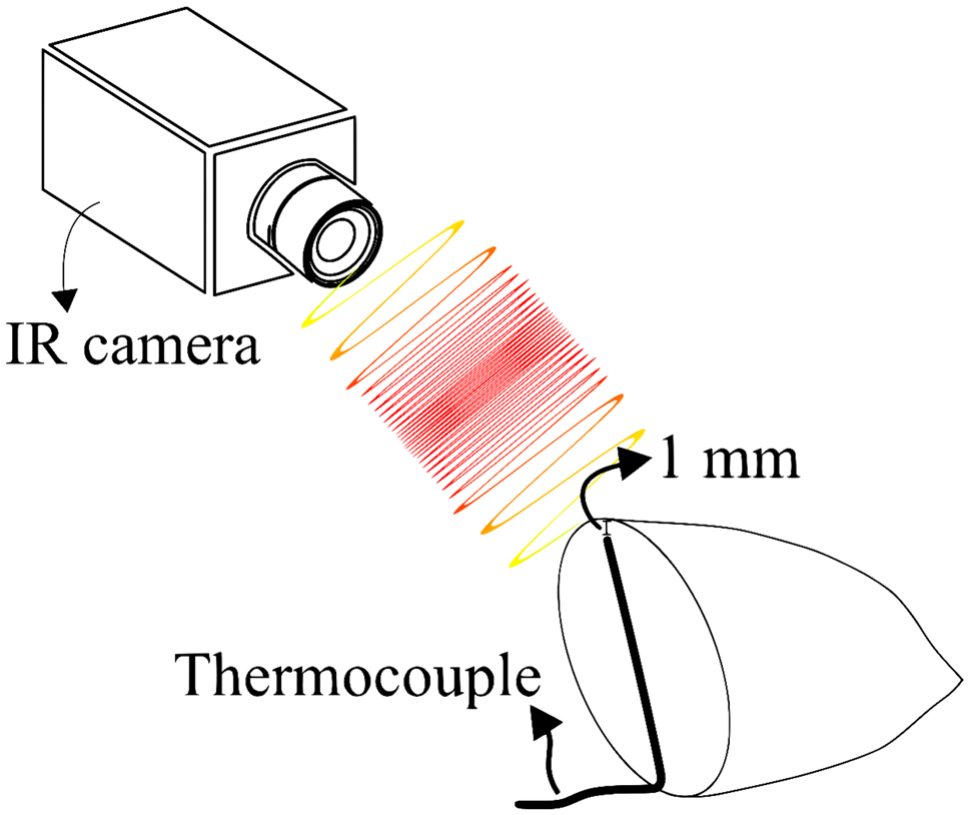
Tandem infrared-thermocouple temperature monitoring system

Preliminary experiments were conducted to determine the maximum treatment time, which did not affect the quality of the treated almonds. The change in color of fresh almond kernels was visually assessed; images were taken at different PL preliminary treatment conditions (Fig. 2). Samples were placed on a shelf at 14.10 cm from the central axis of the UV lamp.

The design of experiments (DoE) is shown in Table 2; five different treatment times were tested for each system configuration. All studied system configurations for the PL inactivation were tested in triplicate; the experimental sequence started from the longest to the shortest treatment time.

**Table 2:** Design of experiments (DoE) for the PL treatments on almond kernel

Power (W)	Frequency (Hz)	Treatment times (s)				
1500	1.8	40	30	20	10	5
	3.0	60	50	40	30	20
	100.0	160	100	70	40	20
1250	1.8	100	80	60	40	20
	3.0	100	80	60	40	20
	100.0	220	160	130	100	80
1000	1.8	160	100	70	40	20
	3.0	160	130	100	60	40
	100.0	250	200	160	130	100

The treatment chamber was cleaned with 70% *v/v* ethanol solution to remove potential cross-contamination between each of the reported tests of the DoE, and the PL tests were performed, maintaining samples at the central position in the chamber to avoid variations in the fluence levels. Hence, each test was performed treating one inoculated almond at the time in the maximum energy point. Furthermore, a custom-made holder made up of a washer held by screws was used to hold the almond in place during the treatment to avoid the displacement of samples due to the equipment’s vibration.

#### Colony-Forming Unit Quantification

After the PL treatment, samples were removed from the chamber using a sterile disposable tweezer, and they were placed in a sterile 50-mL falcon tube along with a 10 mL aliquot of PBS solution. Falcon tubes containing the samples were vortexed for 1 min to dislodge the cells from the almond’s surface and to guarantee the resuspension of the after-treatment surviving cells into the PBS solution. This suspension was first tenfold serially diluted using 9 mL of 2% *w/v* of buffered peptone water (Sigma-Aldrich GranuCult BPW, St. Louis, USA), having three different dilutions to ensure colony numbers within a countable range. Then, 100 µL aliquots were spread plated in duplicate on TSAYE agar petri dishes, subsequently incubated for 24 h at 37 °C. After incubation, the number of survivor colonies was enumerated for each case. Control measurements were also performed using this colony count protocol for non-PL-treated almond samples.

#### Tandem Temperature Measuring System

**Fig. 3:**
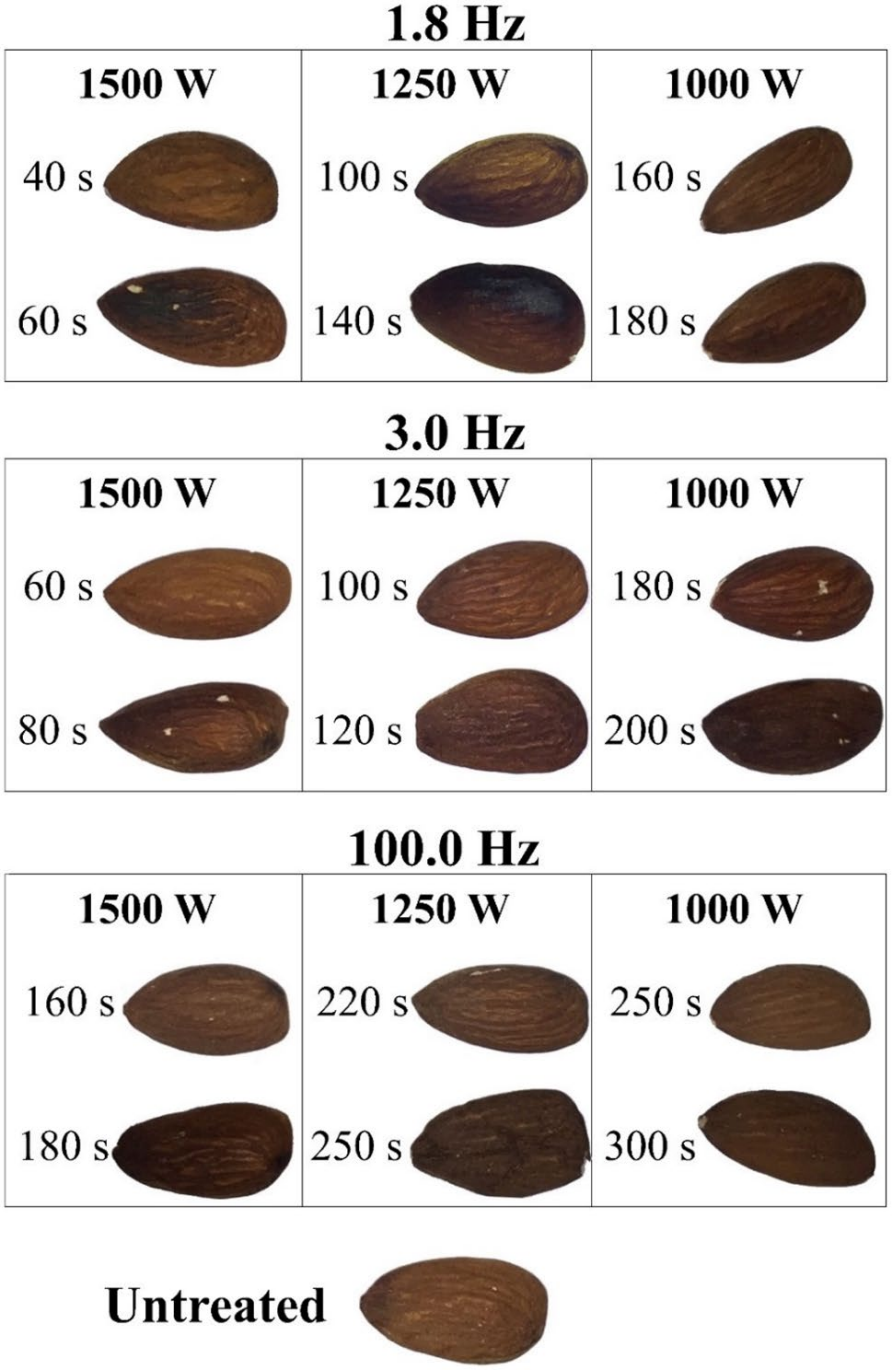
Almond visual appearance after different treatment times. For each condition, the first listed treatment time is the maximum one used for that system configuration of frequency and power. When using the second treatment time presented, almond surface burn is observed; thus, that treatment time was not used in the DoE

Whereas PL is generally regarded as a non-thermal and low-depth penetration treatment, photothermal effects might be present, and they might contribute to microbial inactivation. To evaluate the temperature changes on the almond’s surface, a tandem temperature measuring system was employed. The system evaluated the temperature changes in the food matrix during the application of PL under the selected DoE conditions. The system consisted of two sensors (see Fig. 3):

(i) An infrared camera to collect the surface temperature of the samples (FLIR Systems ThermaCAM™ SC1000, Wilsonville, USA), and it uses a dedicated software (ThermaCAM™ Researcher 2001, Wilsonville, USA) to collect temperature measures each second. Using *ε* = 0.94, adapted from hazelnut reference emissivity of *ε* = 0.95 (Ciarmiello et al., 2013), and,
(ii) A T-type thermocouple placed into an *ad hoc* drilled hole in the almond kernels. It was placed using a 0.508-cm-diameter drill, up to the depth of 1 mm below the surface of the sample that recorded temperature data at 1-s intervals using a data acquisition/switch unit (Keysight Technologies 34970A Agilent, Santa Rosa, USA).

#### Kinetics of Microbial Inactivation and Tested Models for PL Treatments

The quantification of surviving microorganisms after the exposure to lethal agents (e.g., heat, radiation, chemicals, or high-pressure conditions) is typically performed by following their exponential decrease with time. For constant treatment conditions (e.g., isothermal treatments), the phenomenon tends to be satisfactorily described by first-order models, considering similarly susceptible cells subjected to stochastically occurring lethal events over the exposure time. However, since the agent under study was PL, the kinetics of microbial inactivation was analyzed in terms of the fluence (*F*_0_), as recommended in (Gómez-López & Bolton, 2016) (i.e., see the general equation, Eq. 3).

3 $$\frac{\delta N}{\delta t}=-{k}_{i}({F}_{0})\times {{F}_{0}}^{n ({F}_{0})}$$

Four different models were tested to fit the microbial inactivation resulting from the applied PL treatments (Eqs. 4–7):

(i) The log-linear model (i.e., Bigelow and Esty):
  4 $$\mathrm{log}\left(\frac{N}{{N}_{0}}\right)=(\frac{-{k}_{\mathrm{max}}}{\mathrm{ln}10})\times {F}_{0}$$
(ii) The log-linear model with tailing:
  5 $$\mathrm{log}\left(N\right)=\mathrm{log}\left({10}^{\mathrm{log}\left({N}_{0}\right)}-{10}^{\mathrm{log}\left({N}_{\mathrm{res}}\right)}\right){\times e}^{-{k}_{\mathrm{max}}\times {F}_{0}}+{10}^{\mathrm{log}({N}_{\mathrm{res}})}$$
(iii) The one-parameter Weibull model:
  6 $$\mathrm{log}\left(\frac{N}{{N}_{0}}\right)={\left(\frac{-{F}_{0}}{\delta }\right)}^{p}$$
(iv) The two-parameter Weibull model:
  7 $$\mathrm{log}\left(N\right)=\mathrm{log}({N}_{0})\times \mathrm{log}\left(\frac{{10}^{{\left(-\frac{F}{{\delta }_{1}}\right)}^{P}+\alpha } + {10}^{{\left(-\frac{F}{{\delta }_{2}}\right)}^{P}}}{1+{10}^{\alpha }}\right)$$

Knowing the initial number of cells (*N*_0_, CFU·mL^−1^) and the surviving or residual microorganisms (*N*, CFU·mL^−1^) after the exposure to the lethal agent (*F*_0_, J·cm^−2^), the observed kinetics of microbial inactivation can be fitted to the above-proposed models. The log-linear model (Eq. 4) uses the *k*_max_ parameter (cm^−2^·J) to account for the inactivation rate, as the achieved logarithmic reduction by the application of a *F*_0_ = 1 J·cm^−2^. This model can be extended for inactivation curves that first present a log-linear behavior, followed by a less marked reduction in the residual population as the applied agent increases (i.e., commonly known as tailing). This model uses *k*_max_ (cm^−2^·J) for the description of the log-linear region, while the ${N}_{\mathrm{res}}$ represents the tailing section. The tailing effect implies the existence of a subpopulation that is less affected by the treatment, whether due to its own resistance or to protective effects of the surrounding matrix (Cerf, 1977).

In the one-parameter Weibull model (Eq. 6), the fitting procedure requires two constants: the *δ* (J·cm^−2^), which is the fluence required for the first decimal reduction, and the shape parameter, *p* (dimensionless). Lastly, the two-parameter Weibull model (Eq. 7) also uses the shape parameter (*p*) but assumes that the microbial population is constituted by two subpopulations exhibiting different resistances to the inactivation agent. Each of these subpopulations are hence characterized by specific fluence constants *δ*_1_ and *δ*_2_ (in J·cm^−2^), to achieve the first decimal reduction of the specific subpopulation. The last parameter of this model is *α* (dimensionless), which accounts for the ratio between the initial microbial logarithmic concentration of the more sensitive to the less sensitive populations to the lethal agent.

A custom MATLAB code (The MathWorks Inc., R2019b) was developed to analyze the obtained microbial inactivation data, using the proposed kinetics models of Eqs. 4–7. The code used the built-in curve fitting toolbox to fit the model parameters to each case automatically; initial seed parameters were given in the expected range of the models. The kinetics of inactivation for each selected condition of the DoE (Table 2) was performed using triplicate measurements, while the goodness-of-fit for each model was scored by means of two indicators: (i) the root mean sum of squared errors (RMSE) and (ii) the coefficient of determination (*R*^2^).

#### Statistical Analysis

The statistical significance was evaluated using MiniTab 17. One-way ANOVA followed by Tukey pairwise tests were done to verify the independent significance of frequency and power conditions in the resulting inactivation. An additional two-way ANOVA was also performed (Tukey pairwise) to verify the significance of their combined effect. Additional details are provided in the Appendix as supplementary material.

### Results and Discussion

#### Effect of Operating Parameters

The DoE included the inactivation evaluation through different rate frequencies using different control modules. The shown data indicate that higher levels of inactivation can be achieved at lower frequencies; for example, at 1.8 Hz, the achieved logarithmic reductions lie in the 0.68–6.02 range, while for 3.0 Hz, the range is 1.88–4.69 and for 100.0 Hz in the 0.96–3.66, see Table A4 (Appendix). As a matter of fact, the tested lower frequencies (i.e., 1.8 and 3.0 Hz) achieve higher average logarithmic reductions compared to the 100.0-Hz module, considering the experimental uncertainties of the case. These results are consistent with previous findings using led PL in which lower frequencies result in higher inactivation levels (Gillespie et al., 2016; Kim & Kang, 2018; Li et al., 2010).

The effect of the frequency can be explained based on the energy density (i.e., J·cm^−2^·pulse^−1^) delivered in each case by the PL treatment. Since the energy density is inversely proportional to the frequency, a higher microbial inactivation seems achievable by reducing the frequency and providing fewer pulses (at constant treatment time) at higher energy density. However, low PL frequencies might pose a greater risk for the stability of the food matrix (in this case, burning the almond surface) in shorter times. The effect of the frequency should be experimentally tested since it can result in different interactions between the delivered energy and the treated matrix. Certainly, photothermal effects could vary at different frequencies due to changes in heating and after-action periods, the maximum surface temperature, and the thermal penetration depth. Similarly, photophysicochemical effects at different energy densities can reach compromising threshold levels of key structures in the biotic phase (e.g., nucleic acids or cell membranes). For example, significant reversible damages have been found for lower frequencies (20 Hz), compared to larger frequencies (100 Hz) or continuous light (Kim & Kang, 2018).

The effect of the fluence (*F*_0_) should be seen as a combination of factors, the chosen treatment times (*t*), and the delivered specific power (*E*_0_). Following the reciprocity or Bunsen-Roscoe law (BRL), the photochemical effects on a biotic phase tend to be directly proportional to the total applied fluence dose. BRL has also been hypothesized to be valid for PL applications (Galanakis, 2019; Horwitz et al., 1990). Since the *F*_0_ = *E*_0_ × *t*, the extent of photochemical effects is theoretically determined by cumulative applied dose on the almond surface, and it should be linear in respect to the application time (at constant specific power).

Longer fluence doses result in higher inactivation levels, as shown by the one-way ANOVA (Appendix) for all evaluated modules comparing the concurrent treatment times (*p* < 0.05). Moreover, to verify the BRL, a linear regression of the inactivation points for all system configurations is performed in terms of fluence. The data points are dispersed and roughly follow a linear trend (Figure A1, Appendix). However, the linear regression coefficients show a low goodness-of-fit (*R*^2^ = 0.52) evidencing that other factors play a role in the observed CFU-log reductions. The BRL is primarily used for photochemical effects on the biotic phase. In the present case, photothermal effects (see “PL Thermal Analysis” section) might also contribute to the surface inactivation on the food matrix, thus the observed deviations. The effectiveness of PL treatments and the relative extent of the structural/chemical (photophysicochemical) and thermal damages (photothermal) strongly depend on the type of microorganisms, the food matrix’s nature, and the experimental and instrumental setup. Notwithstanding for practitioners and process design, a low time-consuming and a practical approach must be provided for the analysis of inactivation data. Hereinafter, a kinetic study is performed to describe the PL inactivation in the studied systems.

In this study, the treatment times used were restricted by the visual evidence of burning the almond kernel surfaces. Although the visual inspection is part of the recommended guidelines by the USDA for assessing almond quality (United States Department of Agriculture, 1997), colorimetric measures may also be performed (Wiktor et al., 2019). Future studies aiming to further explore changes in quality attributes after PL treatments may assess them by analytical tests such as peroxide level, conjugated diene (Liu et al., 2021), and total antioxidant (Wiktor et al., 2019). Additionally, sensory tests are helpful for novel non-thermal processes. Prakash et al. (2010) found that large irradiation doses (5.25 kGy) rendered the almonds unacceptable, thus this method requires more research to properly eliminate *Salmonella* from raw almonds.

#### Kinetics of Microbial Inactivation by Means of PL

**Fig. 4:**
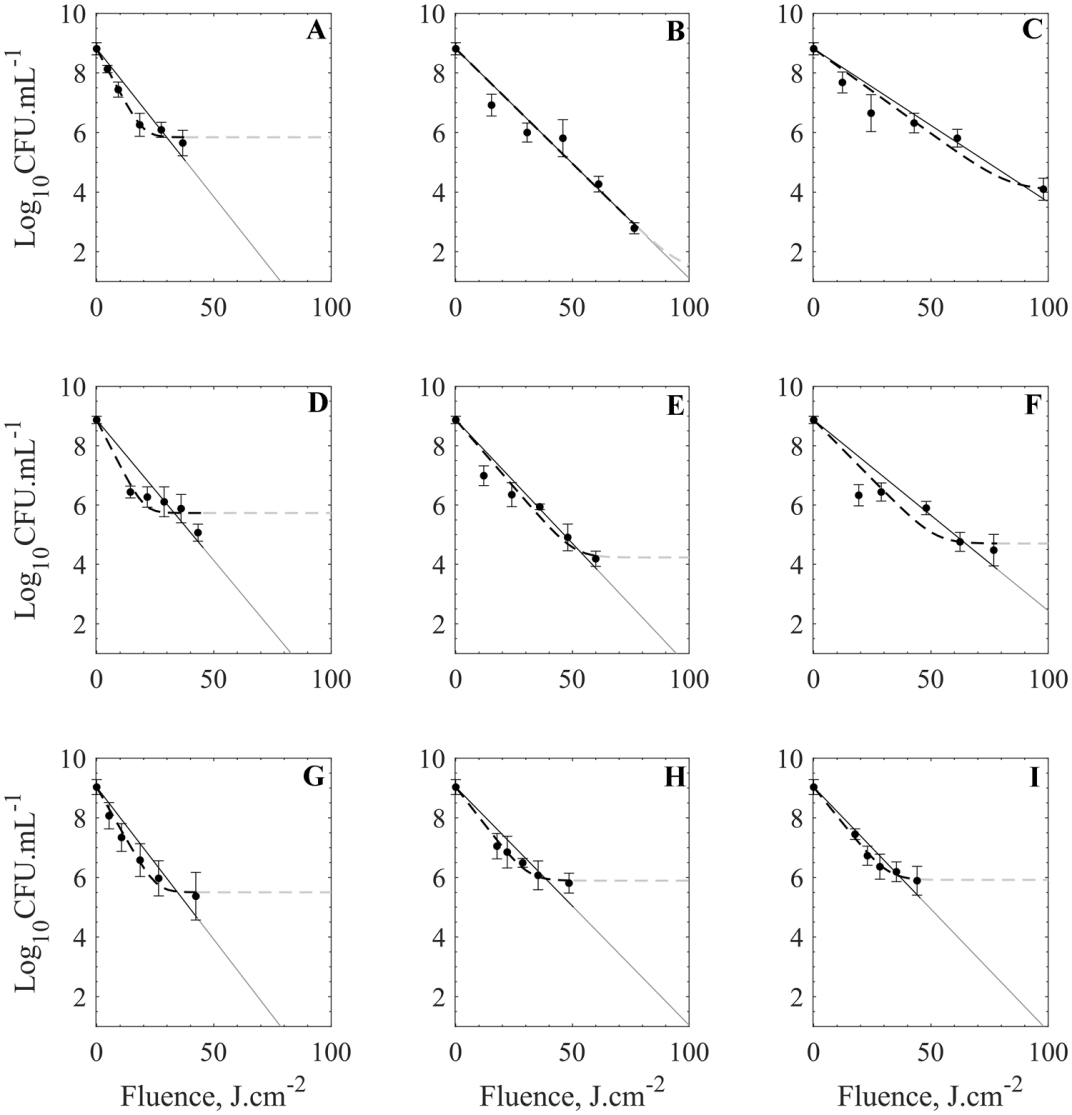
Log-linear (-) and log-linear + tail (–) models plotted together with the survival data and their corresponding uncertainties given as one standard deviation. Data for the 1.8-Hz system (A–C), 3.0 Hz (D–F), and 100.0 Hz (G–I). Input power conditions of 1500 W (A, D, and G), 1250 W (B, E, and H), and 1000 W (C, F, I). Gray lines indicate extrapolated interval

**Fig. 5:**
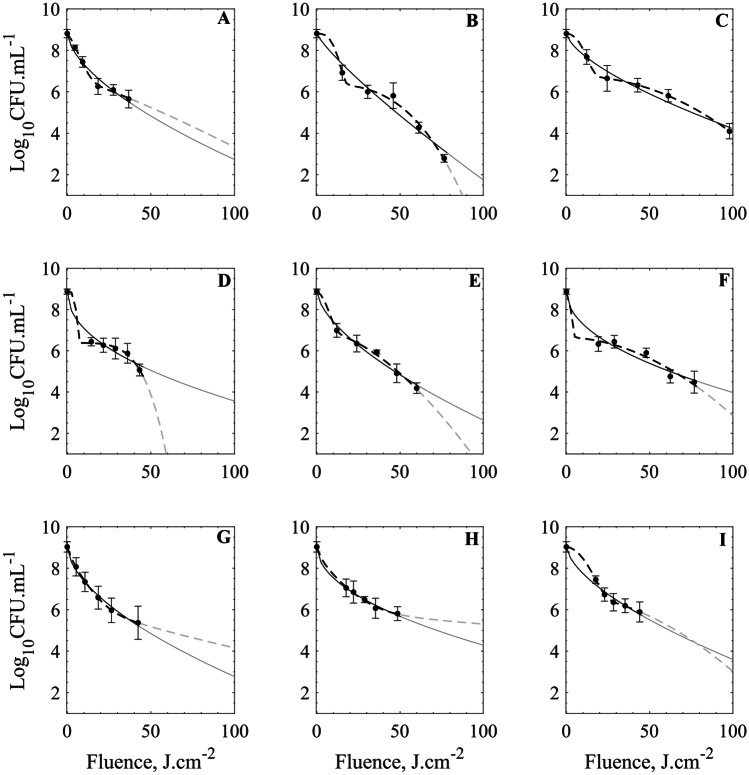
One (-) and two-parameter (–) Weibull models plotted together with the survival data and their corresponding uncertainties given as one standard deviation. Data for the 1.8-Hz system (A–C), 3.0 Hz (D–F), and 100.0 Hz (G–I). Input power conditions of 1500 W (A, D, and G), 1250 W (B, E, and H) and 1000 W (C, F, I). Gray lines indicate extrapolated interval

Significant reductions in the microbial counts are observed after the application of different PL treatments. The survival data are displayed along with their respective experimental uncertainties. These data are presented together with the proposed models to describe the microbial inactivation as a function of the received fluence dose. Two sets of mathematical models are tested; the fitting following the log-linear and log-linear with tailing is displayed in Fig. 4, while the corresponding data for one-parameter Weibull and two-parameter Weibull are presented in Fig. 5.

There are different mathematical approaches to model and interpret the survival curves obtained from bacterial inactivation treatments using either physical or chemical agents. Consistent with reported data for PL inactivation treatments on different food matrices, the log-reduction behavior in the present case seems to deviate from the typical linearity observed for isothermal treatments. Non-traditional inactivation methods tend to yield survival curves that deviate from the log-linear model (i.e., represented in Eqs. 3 and 4). Hence, survival curves might present shoulders or tailing sections for these systems, depending on the specific applied dose and the application conditions. Whereas shoulders in these curves serve to account for the smooth initiation of the inactivation process, the tailing section serves to represent the saturation point of the applied agent (if present).

In this study, four different models are selected: the log-linear, the log-linear with tailing, the one-parameter Weibull, and the two-parameter Weibull (see “Kinetics of Microbial Inactivation and Tested Models for PL Treatments” section, Eqs. 4–7). These models are chosen since they have proved their usefulness to fit microbial inactivation data derived from the application of non-traditional agents, such as pulsed light (Hwang et al., 2019; Izquier & Gómez-López, 2011), high-pressure processing (HPP) (Buzrul, 2017; Yuan et al., 2016), and pulsed electric field (Soliva-Fortuny et al., 2006). Their goodness-of-fit is evaluated statically (see “Kinetics of Microbial Inactivation and Tested Models for PL Treatments” section).

To compare the performance of PL inactivation under the different conditions, the characteristic parameters of each model are presented in Table 3 as well as the correspondent goodness-of-fit. The log-linear model shows the lowest-performing correlation coefficients (i.e., *R*^2^ in the 0.79–0.95 and RMSE in the 0.69–0.33 range), which indicates that the fitting capacity of such model to describe the observed PL microbial inactivation is low. However, the main parameter of this model (*k*_max_) exhibits an increasing trend with the power (at constant frequency conditions), while at different frequencies, the obtained *k*_max_ for each power condition seems almost constant. In practical terms, this model provides a first insight into the inactivation behavior of the PL treatment. The *k*_max_ parameter serves to account for the proportional log-CFU inactivation achieved by applying a unitary dose of the lethal agent (J·cm^−2^). Conversely, the log-linear model with tailing presents improved fitting capacity, achieving higher *R*^2^ values (0.87–0.99) and lower RMSE values (0.63–0.11). In this model, the *k*_max_ describes the inactivation rate in the log linear region and follows a trend like the previous model, but higher values are obtained in the fitting process (see Table 3). The tailing region is considered in the model by incorporating the *N*_res_ parameter that accounts for a residual population which implies a saturation effect of the lethal agent. In fact, this fitted parameter reflects the asymptotic behavior of the survival curves attained with the highest tested doses (longest treatment times).

**Table 3:** Best-fitting parameters for all tested models

*f*	*P*		Log-linear			Log-linear + tail				One-parameter Weibull				Two-parameter-Weibull					
		*log*_10_(*N*_0_)	*k*_max_	RMSE	*R*^2^	*N*_res_	*k*_max_	RMSE	*R*^2^	*δ*	*s*	RMSE	*R*^2^	*δ*_1_	*s*	*α*	*δ*_*2*_	*RMSE*	*R*^*2*^
1.8 Hz	1500 W	8.81	0.23	0.45	0.87	5.84	0.34	0.15	0.99	5.23	0.61	0.24	0.97	6.67	1.21	2.10	36.44	0.10	1.00
	1250 W	8.81	0.18	0.46	0.95	1.40	0.18	0.51	0.95	9.66	0.84	0.46	0.96	12.02	2.90	2.44	49.03	0.25	0.99
	1000 W	8.81	0.12	0.50	0.90	4.07	0.13	0.50	0.92	9.85	0.66	0.26	0.98	11.33	2.15	2.02	61.89	0.01	1.00
3.0 Hz	1500 W	8.87	0.22	0.58	0.79	5.73	0.36	0.44	0.91	2.96	0.47	0.23	0.97	5.28	4.53	2.49	40.82	0.09	1.00
	1250 W	8.87	0.19	0.48	0.91	4.23	0.21	0.47	0.93	5.42	0.63	0.23	0.98	7.21	1.69	1.86	32.18	0.14	1.00
	1000 W	8.87	0.15	0.69	0.81	4.70	0.18	0.63	0.87	3.95	0.49	0.33	0.96	2.74	1.89	2.22	49.50	0.33	0.98
100.0 Hz	1500 W	9.03	0.24	0.53	0.85	5.50	0.32	0.21	0.98	4.26	0.58	0.15	0.99	5.38	0.77	2.42	31.19	0.03	1.00
	1250 W	9.03	0.18	0.45	0.85	5.89	0.23	0.14	0.99	4.45	0.50	0.08	1.00	6.43	0.67	2.77	106.91	0.08	1.00
	1000 W	9.03	0.19	0.33	0.92	5.92	0.23	0.11	0.99	6.68	0.63	0.19	0.98	13.53	1.85	2.32	49.41	0.02	1.00

The one-parameter Weibull model presents an increased fitting capacity with higher *R*^2^ and lower RMSE values. For each fitting, the shape parameter remains consistent with an *s* < 1 (concavity) along all tested conditions. The other parameter, *δ*, presents an inverse relation with the power conditions. That is, higher power (1500 W) tends to yield lower sigma values. This is probably since the lethal agent, the fluence, interacts differently with the biotic phase at different power conditions, which changes the required dose for a logarithmic reduction.

The last tested model, the two-parameter Weibull, shows the best goodness-of-fit, either in terms of the *R*^2^ (> 0.98) or the RMSE (< 0.33) coefficients. The *α* parameter, which is related to the microbial systems under analysis and the effectiveness of the lethal agent, is consistent for all tested conditions (1.9–2.8). The value of this parameter indicates threshold resistance differences to the applied treatment. For example, *α* = 1 indicates lethality uniformity (either understood as no different subpopulations or different deactivation mechanisms playing a role), while higher values support the multiple subpopulations or resistance hypotheses. This fact is also reflected in the *δ*_1_ and *δ*_2_ values; larger differences between these quantities suggest higher resistance differences during the treatment. Nonetheless, the obtained values lie in wide ranges, 2.74–13.53 and 31.19–106.91 for *δ*_1_ and *δ*_2_, respectively.

#### PL Thermal Analysis

**Fig. 6:**
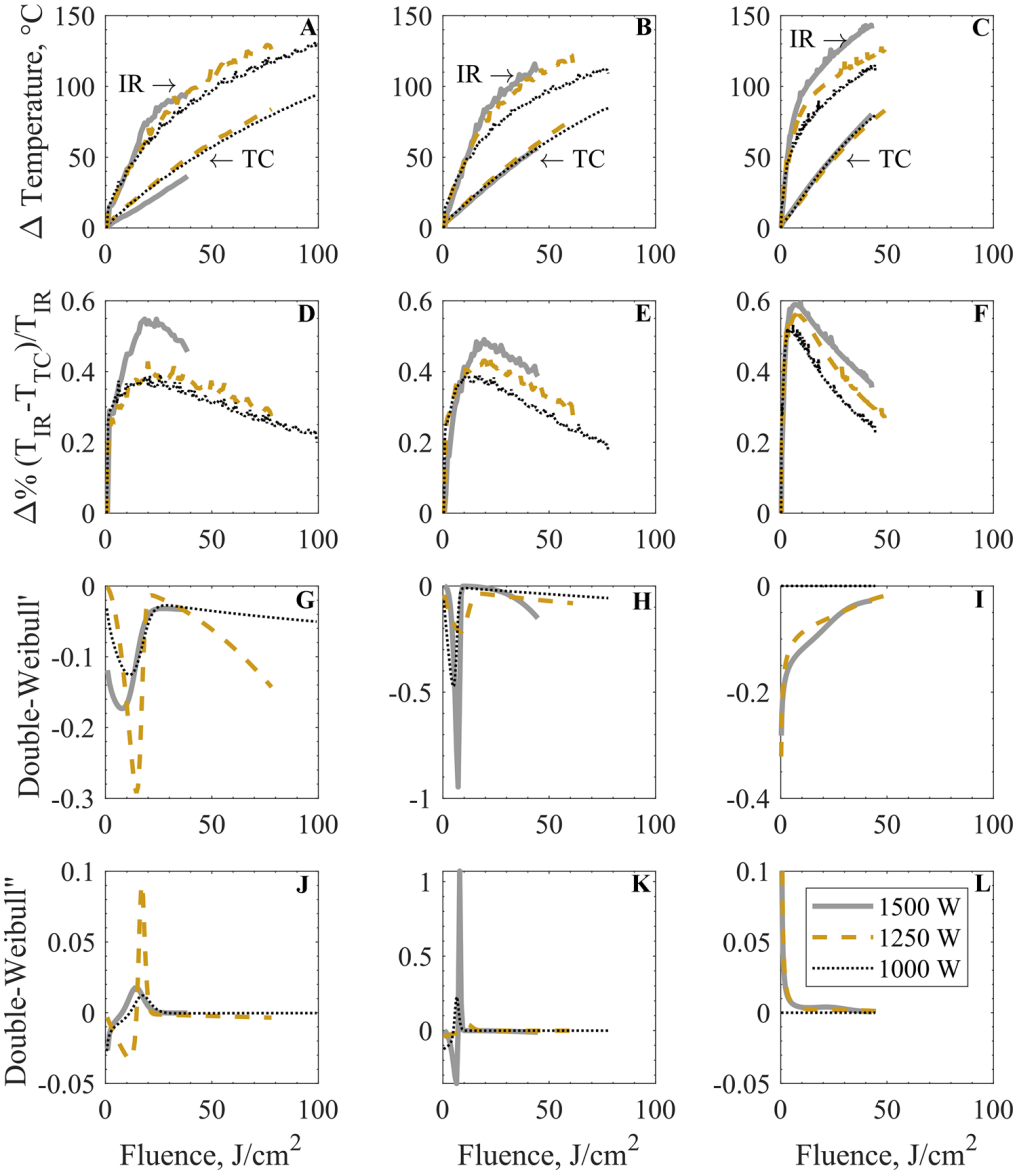
Thermal analysis. IR and thermocouple temperature profiles (A–C) and the percentual change in the offset between IR and TC (D–F). Then, the first (G–I) and the second (J–L) derivative of the two-parameter Weibull model are shown. Lines represent data for the 1500 W (-), 1250 W (–), and 1000 W (..). Graphs in the first column (A, D, G, and J) are for the 1.8-Hz system, in the second column (B, E, H, and K) are for the 3.0-Hz system, and on the third column (C, F, I, and L) are for the 100.0-Hz system

Considering the higher fluence doses used in this study, a thermal analysis is in order. There are several factors that play an important role in the overall heat balance. Heat fluxes can change relevance throughout the treatment. Figure 6 depicts both the temperature measurements of the IR and the thermocouple readings for all tested experimental conditions (i.e., frequency and power levels). First, a temperature difference can be seen between the surface temperature and the inner superficial layer (where the thermocouple is placed). Besides the difference in the maximum reached values, there is also a difference in the evolution temperature profiles: while the thermocouple presents a rather linear trend with respect to the fluence, the IR profiles present a biphasic behavior. This behavior is characterized by an initial steep linear slope that gradually turns more asymptotic, consistent with the literature reference for similar applications (Bingol et al., 2011; Gao et al., 2011). In the linear phase of the IR profile, the steepness is roughly 2–3 times higher than the one from the thermocouple readings. This fact probably reflects the different heating phenomena and fluxes which are measured at each point: while the thermocouple primarily reads the result of conductive mechanisms within the almond, the IR also encompasses convective and radiative components at the surface.

Additionally, the offset between the two measurements and the percentual differences (using the IR measurements as reference) is calculated and displayed in Fig. 6. The abovementioned heat transfer considerations can also be observed in these charts, where the initial offsets are modest, in the order of 2 °C, corresponding to roughly a 10% difference. The transient behavior is also observed in the offset evolution, which then stabilizes, reaching a pseudo-stationary state.

The temperature measurements can be related to the observed PL microbial inactivation. In particular, Fig. 6, where the maximum offset between the two collected temperatures is presented. The reached maximum point lies in a similarly narrow range for all tested power conditions at 1.8 and 3.0 Hz, which is 18–20 J·cm^−2^ for the former and 17–18 J·cm^−2^ for the latter. Conversely, the system at 100.0 Hz reaches a maximum for the offset temperatures in the 8–10 J·cm^−2^. These maxima offset readings correspond to the highest achieved inactivation rates, given by the first derivative of the best-fitting model (the two-parameter Weibull as shown in Fig. 6 G-I). After the maxima inactivation rates are achieved, a slowdown in the rates is observed. Indeed, the second derivates (Fig. 6 J-L) are also consistent with the reported linear decreases (offset temperature after the maxima, Fig. 6 D-F).

### Conclusions

In the present work, *S. enteritis* PL inactivation treatments on the almond surface are investigated. The different frequencies and power conditions used for the treatments indicated that up to 6 CFU-log reductions could be achieved (1.8 Hz, 1250 W, and 100-s treatment time), without noticeable impairment of the quality of the product. The fluence effect should be rationalized in terms of optimal frequency and power conditions to reach desired inactivation levels without compromising the food matrix. Low frequencies and moderate power conditions seem to be an adequate starting point for optimization. Contrary to traditional isothermal inactivation, the present inactivation curves, in terms of the fluence, show nonlinear behavior, and the best suitable model for the trends is the two-parameter Weibull (*R*^2^ > 0.98 for all systems). Although PL is generally considered a non-thermal process, surface heating at high fluence doses should not be neglected. Higher inactivation rates are observed coupled to surface heating (IR), while reduced inactivation rates are evinced for more stationary surface temperature profiles. Hence, thermal monitoring proved insightful to elucidate whether the rate of inactivation could be linked to the surface thermal dynamics. This combined approach, using mathematical models and temperature monitoring, represents a promising method for studying and implementing novel inactivation techniques, in particular regarding photothermal effects.

## Supplementary Information

Below is the link to the electronic supplementary material.

Supplementary file1 (DOCX 37 KB)

## Data Availability

Experiments to acquire the raw data were generated at the Institute for Food Safety and Health, Illinois Institute of Technology, USA. All data processing and intellectual work were done at Politecnico di Torino, Italy. Additional information can be found in the Appendix with supporting information. Any additional data supporting the findings of this study are available from the corresponding author upon request.
