# Supplementary material for: Pulsed Light (PL) Treatments on Almond Kernels: Salmonella enteritidis Inactivation Kinetics and Infrared Thermography Insights
Source: Food Bioproc Tech. 2021 Nov 4;14(12):2323–35. doi: 10.1007/s11947-021-02725-9 (PMC8566968; doi:10.1007/s11947-021-02725-9)
Supplement: Supplementary file 1 — Supplementary file1 (DOCX 37 KB) [file 11947_2021_2725_MOESM1_ESM.docx]

**Experimental Pulsed Light (PL) treatments on** **almond kernels: *Salmonella Enteritidis* inactivation kinetics and infrared thermography insights**

Maitê Harguindeguy and Carlos E. Gómez-Camacho

*DISAT, Dep. Applied Science and Technology, Politecnico di Torino, C/so duca degli Abruzzi 24, 10129 Torino, Italy*

**APPENDIX**

*Fluence tables*

Tables A1, A2 and A3 show the corresponding applied fluences based on the 14.5 cm distance, system frequency and power used and assuming that 40% of the applied light is in the UV region (Huang and Chen 2014).

**Table A1.** Fluences for each treatment condition for the 1.8 Hz system.

| Power, W | Duration, s | Fluence dose applied, **J·cm^-2^** |
| --- | --- | --- |
| 1500 | 5 | 5 |
| 1500 | 10 | 9 |
| 1500 | 20 | 18 |
| 1500 | 30 | 28 |
| 1500 | 40 | 37 |
| 1250 | 20 | 15 |
| 1250 | 40 | 31 |
| 1250 | 60 | 46 |
| 1250 | 80 | 61 |
| 1250 | 100 | 77 |
| 1000 | 20 | 12 |
| 1000 | 40 | 24 |
| 1000 | 70 | 43 |
| 1000 | 100 | 61 |
| 1000 | 160 | 98 |

**Table A2.** Fluences for each treatment condition for the 3.0 Hz system.

| Power, W | Duration, s | Fluence dose applied, **J·cm^-2^** |
| --- | --- | --- |
| 1500 | 20 | 14 |
| 1500 | 30 | 22 |
| 1500 | 40 | 29 |
| 1500 | 50 | 36 |
| 1500 | 60 | 43 |
| 1250 | 20 | 12 |
| 1250 | 40 | 24 |
| 1250 | 60 | 36 |
| 1250 | 80 | 48 |
| 1250 | 100 | 60 |
| 1000 | 40 | 19 |
| 1000 | 60 | 29 |
| 1000 | 100 | 48 |
| 1000 | 130 | 62 |
| 1000 | 160 | 77 |

**Table A3.** Fluences for each treatment condition for the 100.0 Hz system.

| Power, W | Duration, s | Fluence dose applied, **J·cm^-2^** |
| --- | --- | --- |
| 1500 | 20 | 5 |
| 1500 | 40 | 11 |
| 1500 | 70 | 19 |
| 1500 | 100 | 26 |
| 1500 | 160 | 42 |
| 1250 | 80 | 18 |
| 1250 | 100 | 22 |
| 1250 | 130 | 29 |
| 1250 | 160 | 35 |
| 1250 | 220 | 49 |
| 1000 | 100 | 18 |
| 1000 | 130 | 23 |
| 1000 | 160 | 28 |
| 1000 | 200 | 35 |
| 1000 | 250 | 44 |

*Statistical analysis for the system averages*

**Table A4.** One-way ANOVA results for the effect of frequency in the achieved inactivation.

| **Frequency** | **N** | **Mean** | **Grouping** |
| --- | --- | --- | --- |
| 3 | 50 | 3.1 | A |
| 2 | 54 | 2.9 | A |
| 100 | 50 | 2.6 | A |

Obs: p-value = 0.098.

**Table A5.** One-way ANOVA results for the effect of power in the achieved inactivation.

| **Power** | **N** | **Mean** | **Grouping** |
| --- | --- | --- | --- |
| 1250 | 52 | 3.2 | A |
| 1000 | 51 | 3.0 | A |
| 1500 | 51 | 2.5 | B |

Obs: p-value = 0.004.

**Table A6.** Two-way ANOVA results for the combined effect of power and frequency in the achieved inactivation.

| **System** | **N** | **Mean** | **Grouping** |
| --- | --- | --- | --- |
| 1.8Hz 1250W | 19 | 3.5 | A |
| 3Hz 1000W | 16 | 3.3 | A |
| 3Hz 1250W | 16 | 3.1 | A B |
| 1.8Hz 1000W | 19 | 3.1 | A B |
| 3Hz 1500W | 18 | 2.8 | A B |
| 100Hz 1250W | 17 | 2.7 | A B |
| 100Hz 1000W | 16 | 2.7 | A B |
| 100Hz 1500W | 17 | 2.5 | A B |
| 1.8Hz 1500W | 16 | 2.1 | B |

Obs: For power, p-value = 0.003, for frequency p-value = 0.077.

*Statistical analysis by system configuration*

**Table A7.** One-way ANOVA results for fluence doses applied using the 1.8 Hz system.

|  | **Dose, J·cm^-2^** | **N** | **Mean Log-CFU reduction** | **Mean Grouping** |
| --- | --- | --- | --- | --- |
| **1500 W** | 40 | 4 | 3.1 | A |
|  | 30 | 3 | 2.6 | AB |
|  | 20 | 3 | 2.3 | B |
|  | 10 | 3 | 1.3 | C |
|  | 5 | 3 | 0.6 | C |
| **1250 W** | 100 | 3 | 6.0 | A |
|  | 80 | 4 | 4.6 | B |
|  | 60 | 5 | 3.0 | C |
|  | 40 | 4 | 2.8 | C |
|  | 20 | 3 | 1.6 | D |
| **1000 W** | 160 | 3 | 4.6 | A |
|  | 100 | 3 | 2.9 | B |
|  | 70 | 3 | 2.4 | BC |
|  | 40 | 3 | 2.1 | CD |
|  | 20 | 3 | 1.3 | D |

**Table A8.** One-way ANOVA results for fluence doses applied using the 1.8 Hz system.

|  | **Dose, J·cm^-2^** | **N** | **Mean Log-CFU reduction** | **Mean Grouping** |
| --- | --- | --- | --- | --- |
| **1500 W** | 43 | 3 | 3.8 | A |
|  | 36 | 4 | 3.0 | AB |
|  | 29 | 4 | 2.6 | B |
|  | 22 | 4 | 2.5 | B |
|  | 14 | 3 | 2.4 | B |
| **1250 W** | 60 | 3 | 4.7 | A |
|  | 48 | 3 | 4.0 | A |
|  | 36 | 3 | 2.9 | B |
|  | 24 | 4 | 2.5 | BC |
|  | 12 | 3 | 1.8 | C |
| **1000 W** | 77 | 4 | 4.4 | A |
|  | 62 | 3 | 3.9 | A |
|  | 48 | 3 | 3.0 | B |
|  | 29 | 3 | 2.5 | B |
|  | 19 | 3 | 2.5 | B |

**Table A9.** One-way ANOVA results for fluence doses applied using the 1.8 Hz system.

|  | **Dose, J·cm^-2^** | **N** | **Mean Log-CFU reduction** | **Mean Grouping** |
| --- | --- | --- | --- | --- |
| **1500 W** | 42 | 2 | 4.7 | A |
|  | 42 | 1 | 3.8 | AB |
|  | 26 | 4 | 3.1 | BC |
|  | 19 | 4 | 2.5 | BC |
|  | 11 | 3 | 1.8 | CD |
| **1250 W** | 5 | 3 | 1.0 | D |
|  | 35 | 3 | 2.9 | AB |
|  | 29 | 3 | 2.7 | AB |
|  | 22 | 4 | 2.5 | AB |
|  | 18 | 3 | 2.1 | B |
| **1000 W** | 44 | 4 | 3.1 | A |
|  | 35 | 3 | 2.9 | AB |
|  | 28 | 3 | 2.7 | AB |
|  | 23 | 3 | 2.4 | AB |
|  | 18 | 3 | 2.0 | B |

*BRL evaluation*

Figure A1. Linear regression using all replications for all testes conditions in terms of fluence.
